# Comparison of germinal center markers CD10, BCL6 and human germinal center-associated lymphoma (HGAL) in follicular lymphomas

**DOI:** 10.1186/1746-1596-6-97

**Published:** 2011-10-11

**Authors:** Gaia Goteri, Guendalina Lucarini, Antonio Zizzi, Antonello Costagliola, Federica Giantomassi, Daniela Stramazzotti, Corrado Rubini, Pietro Leoni

**Affiliations:** 1 Department of Biomedical Sciences and Public Health, Pathological Anatomy, Polytechnic University of Marche Region, Ancona Hospital, Ancona, Italy; 2 Department of Molecular and Clinical Sciences, Histology, Polytechnic University of Marche Region, Ancona, Italy; 3 Department of Molecular and Clinical Sciences, Clinic of Hematology, Polytechnic University of Marche Region, Ancona Hospital, Ancona, Italy

## Abstract

**Background:**

Recently, human germinal center-associated lymphoma (HGAL) gene protein has been proposed as an adjunctive follicular marker to CD10 and BCL6.

**Methods:**

Our aim was to evaluate immunoreactivity for HGAL in 82 cases of follicular lymphomas (FLs) - 67 nodal, 5 cutaneous and 10 transformed - which were all analysed histologically, by immunohistochemistry and PCR.

**Results:**

Immunostaining for HGAL was more frequently positive (97.6%) than that for BCL6 (92.7%) and CD10 (90.2%) in FLs; the cases negative for bcl6 and/or for CD10 were all positive for HGAL, whereas the two cases negative for HGAL were positive with BCL6; no difference in HGAL immunostaining was found among different malignant subtypes or grades.

**Conclusions:**

Therefore, HGAL can be used in the immunostaining of FLs as the most sensitive germinal center (GC)-marker; when applied alone, it would half the immunostaining costs, reserving the use of the other two markers only to HGAL-negative cases.

## Background

Follicular lymphoma (FL) is the commonest form of indolent B-cell lymphomas and the second most frequent type of non-Hodgkin's lymphoma (NHL) in western countries [1]. Biologically, FL represents the neoplastic equivalent of the normal germinal center (GC) reaction, recapitulating the reactive follicles cellular composition [2]. An "in situ" phase is known for FL in which neoplastic cells are confined to GCs [3]; when it becomes invasive, lymphoma expands follicles and extends into the interfollicular areas acquiring variable architectural patterns in which neoplastic follicles can be crowded or spaced apart, irregular, coalesced, serpiginous, confluent, or serrated; they can show mantle or marginal zone differentiation; less frequently they have a predominantly diffuse pattern of growth [4]. FL cells typically express B-cell markers (CD19, CD20, CD22, and CD79a), together with GC-markers (CD10 and BCL6), and have monotypic surface immunoglobulin. At molecular level, they have clonally rearranged, hypermutated immunoglobulin genes [1] and up to 95% contain a translocation between chromosomes 14 and 18, involving immunoglobulin heavy chain (*IGH@*) at 14q32 and *BCL2 *at 18q21 [5-14]. As a consequence of the translocation, FL generally exhibits ectopic *BCL2 *expression.

Recently, human germinal center-associated lymphoma (HGAL) gene protein, also known as germinal center expressed transcript-2 (GCET2), has been proposed as an adjunctive GC-marker which can be detected in benign and malignant follicles [4,15,16]. HGAL has been originally described by Lossos et al. (2003) [15] who identified the gene by conducting a search for genes predicting outcome in large B-cell lymphoma (LBCL). HGAL is a highly evolutionary conserved gene with marked similarity to the mouse GC gene, M17. Evaluated by RT-PCR among normal tissues, a high mRNA expression was found in GC lymphocytes, spleen in humans and mice, compared to other tissues, suggesting a GC-specific gene function. In non-lymphopoietic organs HGAL gene was not expressed, except in lung. HGAL functions as an adaptor protein with a potential regulatory role and mediate some IL-4 effects during physiologic response. IL-4 is a growth factor promoting GC B-cell differentiation into memory B cells and the absence of IL-4 enhances the GC reaction in secondary immune responses. HGAL expression in memory B cells, though at lower levels than in GC lymphocytes, provides indirect support for the involvement of HGAL in the differentiation from GC to memory B cells. Examination of HGAL mRNA expression in malignant cell lines revealed variable expression in B-cell NHL cell lines, minimal expression in Jurkat T cells, and no expression in non-lymphoid HL60 and K562 cell lines. When analyzed in a spectrum of NHL tumor specimens, HGAL expression is high in all FL and low in all chronic lymphocytic leukemia, in all T lymphoblastic lymphoma and in most mantle cell lymphoma (MCL) specimens. A single MCL specimen that demonstrated high HGAL expression, was peculiar for the high content of normal GC. HGAL immunostaining is present in 100% of Burkitt lymphomas, in 87.5% of mediastinal large B lymphomas, and in 68% of DLBCLs and also Hodgkin's lymphomas appeared HGAL positive (70.5% of Lymphocyte-predominant and 72% of classical Hodgkin lymphomas) [16]. Application of HGAL immunostaining is also useful in diagnosing primary cutaneous B-cell lymphomas (CFL) [17] and defining prognosis of diffuse large B-cell lymphomas (DLBCL) [15].

In this study, we analyzed HGAL immunostaining in our series of FL which were all studied histologically, by immunohistochemistry for CD10, BCL6, BCL2 and Ki67-antigen expression, and by PCR for B-cell clonality and *MBR-BCL2/JH *rearrangement.

## Methods

### Pathological samples

Cases with a FL histological diagnosis were selected from the complete list of histological diagnoses performed at the Department of Anatomic Pathology at the University Hospital in Ancona (Italy), between 1996 and 2009. Formalin-fixed and paraffin-embedded tissue blocks were retrieved to perform morphological evaluation and immunohistochemical studies. Overall, the series was composed by 82 cases. The histology was reviewed according to the World Health Organization (WHO) Classification of lymphomas revised in 2008 [2]; the pattern of growth and the histologic grade were defined in each case, except for cutaneous lymphomas. Institutional Review Board approval was obtained for our study.

### Immunohistochemical analysis

Immunohistochemical analyses were performed on paraffin blocks. Conventional 6-μm-thick histological sections were obtained with a microtome and mounted on slides pretreated with poly-l-lysine (Sigma Chemicals, St Louis, MO). Slides were then deparaffinised and rehydrated in ethanol and xylene. Two antigen retrieval methods were applied, 1) by incubating sections with a Tris-EDTA solution, pH 9.0, at 95-98°C for 25 min before staining with antibodies against BCL2, BCL6, CD10, and 2) by incubating sections with 0.01 M citrate buffer solution, pH 6.0, for 15 min before staining with antibodies against Ki67 and HGAL. After washing in H_2_O and then in Tris-buffer solution, sections were incubated with the following monoclonal antibodies: anti-BCL2 (clone 124, dil. 1:100, Dakocytomation, Milan, Italy); anti-BCL6 (clone PG-B6p, dil. 1:10, Dakocytomation, Milan, Italy); anti-CD10 (clone 56C6, dil. 1:30, Novocastra, Laboratories, New Castle, UK); anti-Ki67-antigen (clone Mib-1, dil. 1:80, Dakocytomation, Milan, Italy), and with the polyclonal anti-HGAL (dil. 1:400, Sigma-Aldrich, Milan, Italy). Incubations were carried out in humidified atmosphere at room temperature for 1 hour. The reaction was revealed using the streptavidin-biotin-peroxidase technique (Dako-Envision Plus/HRP peroxidase kit, Dakocytomation, Milan, Italy). Sections were then incubated with 3,3'-diaminobenzidine (0.05 diaminobenzidine in 0.05 M Tris buffer, pH 7.6 and 0.01% hydrogen peroxide; Sigma-Aldrich, Milan, Italy), counterstained with Mayer hematoxylin (Bio-Optica SpA, Milano, Italy) and cover-slipped with Paramount. Reactive tonsil tissue was used as positive controls for GC-markers and staining with omission of the primary antibody was performed as negative control.

The original slides immunostained for commonly used markers in lymphoma diagnostics, like CD20, CD3, CD5, BCL1/Cyclin D1, immunoglobulin light chains, CD21, CD23, were reviewed; when not available or not easily interpreted, the immunostaining was done or repeated.

The number of HGAL, BCL6, BCL2, CD10, and Ki67-antigen immunoreactive cells was counted among at least 500 cells in the more representative fields by using a light microscope at 250× magnification and expressed as percentage. Counting was repeated three times; the obtained mean values were considered for further analysis. For Ki67-antigen a three-point scale was used: low proliferative index for positive values ≤ 25%, intermediate proliferative index for > 25 and ≤ 50%, high proliferative index for values > 50%. HGAL, BCL6, BCL2, and CD10 positivity of immunostaining was then classified with a two-point scale: negative (-) for absence of immunostaining or positivity in less than 20% of positive cells; positive (+) for immunostaining in more than 20%. For BCL2 and BCL6, the immunostaining was considered only in the B-cell population, by comparing it with the CD3 staining in order to avoid the T-cell component marked by BCL2 and by BCL6.

### Molecular studies

Molecular analyses were performed on genomic DNA extracted by paraffin blocks. DNA samples were amplified by PCR according to the already published methods for the FR3 and FR2 immunoglobulin heavy chain gene, for the beta-globin gene and *MBR-BCL2/JH *rearrangement [18-20]. PCR products were run on polyacrilamide gels and visualized by ethidium bromide staining.

### Statistical analysis

Data were entered into the SPSS statistical program (SPPS Inc., Chicago, IL) in a PC. Data were expressed as mean and range for continuous variables and by frequency tabulations for categorical variables. Differences between groups were analyzed by the χ^2 ^test (or Fisher's exact test) for contingency tables and by ANOVA test for numerical variables. Statistical significance was set at p < 0.05.

## Results

Clinical, histological, phenotypical and molecular findings in the present series of FL are summarized in Table 1. The diagnosis of FL was confirmed in all 82 cases retrieved from the archives: 77 were nodal FL, of which 67 were only constituted by FL and 10 showed FL in combination with a LBCL; the last 5 cases were cutaneous FL which appeared negative for extra-cutaneous disease at the diagnosis and during the follow-up and were therefore considered primary CFL.

**Table 1:** Clinical, histological, phenotypical and molecular findings in the series of 82 FL

Gender	F	46
	M	36
Age	Median 58	Range 20-88

Type of FL	Nodal FL	67
	Combined FL+DLBCL	10
	Cutaneous	5

Pattern	"In situ neoplasia"	6 (7.8%)
	Nodular	62 (80.5%)
	Nodular and diffuse	5 (6.5%)
	Diffuse	4 (5.2%)

WHO Grade	Grade 1	8 (11.3%)
	Grade 2	37 (52.1%)
	Grade 3	26 (36.6%)

Marginal differentiation	Yes	7 (9.9%)
	No	64 (90.1%)

BCL6	Positive	76 (92.7%)
	Negative	6 (7.3%)

CD10	Positive	74 (90.2%)
	Negative	8 (9.8%)

BCL2	Positive	72 (87.8%)
	Negative	10 (12.2%)

Ki67 antigen	Low	23
	Intermediate	30
	High	29

*IGH *gene rearrangement	Monoclonal	66 (80.5%)
	Polyclonal	16 (19.5%)

*MBR-BCL2/JH *rearrangement	Present	19 (23.2%)/15 (27.3%)*
	Absent	63 (76.8%)/40 (72.7%)*

* Limited to cases with successful beta-globin gene amplification.

**Figure 1:**
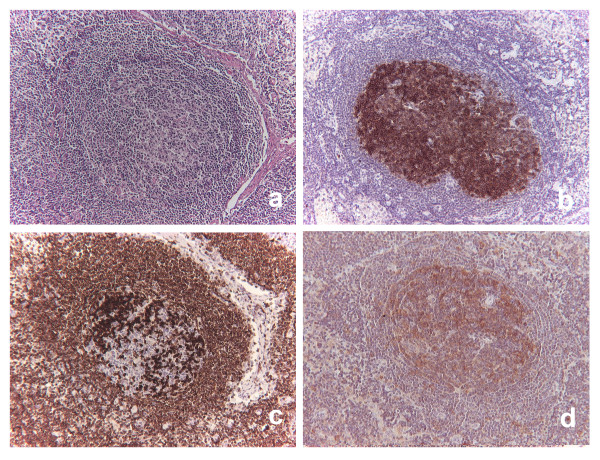
**"In situ" follicular neoplasm**. The follicle depicted in serial sections shows partial polarization loss and prevalence of small cells (a), all staining for CD10 (b), strongly for BCL2 (c) and for HGAL (d).

No difference for age and gender was found among the 3 subgroups ("pure" FL: 28 M, 39F, median age 59 yrs; FL in combination with LBCL: 5 M, 5 F, median age 54 yrs; primary CFL: 3 M, 2 F, median age 53 yrs). Of the nodal FL cases, the most numerous group were those showing grade 2 with a predominantly nodular pattern. Marginal zone differentiation was seen only in this group (7 cases, 9.9%). Six cases showed an "in situ" pattern: in all, the lesion was incidentally discovered; they were 3 males and 3 females, with no difference in age compared to the other subgroups of FL (median age, 61.5, range 43-79). The lesion was diagnosed during the pathological staging of epithelial neoplasms in regional lymph nodes in 4 cases (three women with breast carcinomas and the lesion involving the axillary lymph nodes, and one man with gastric cancer, with the lesion involving the peri-gastric lymph nodes). In the other 2 cases the lesion was found in slightly enlarged inguinal lymph nodes excised during vascular surgery. In all cases, in lymphoid tissue devoid of metastases or any other destructive process, besides normal looking follicles, a variable number of germinal centers, ranging from few to the majority, showed partial or total substitution by small B-lymphoid cells with centrocyte appearance, immunoreactive for CD10 and BCL2 in all cases, for BCL6 in 3 cases, and with a mean Ki67-index of 5%; in all cases the cell infiltrate did not show tendency to invade the interfollicular areas (Figure 1a, b, c). Cytoplasmic HGAL staining was confined to the atypical GCs with a stronger intensity in small atypical cells with strong staining for BCL2 (Figure 1d). The immunostaining for BCL2 was intense and strong in all cases. The difference of Ki67 proliferation index was statistically significant compared to the other invasive FL (ANOVA test, p = 0.003). According to WHO recommendations, patients were staged, but they did not receive any treatment as no evidence of "overt" FL elsewhere was found. By PCR analysis, four cases showed either monoclonal IGH@ gene rearrangement (2) or *MBR-BCL2/JH *rearrangement (2).

**Figure 2:**
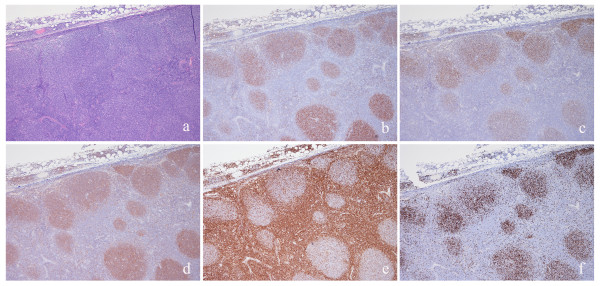
**Invasive follicular lymphoma**. FL is formed by large nodules with interfollicular invasion (a) showing positive staining for CD10 (b), BCL6 (c), HGAL (d); the positivity for GC markers are more evident in the nodular component than in the interfollicular areas in this case. BCL2 was moderately and partially expressed (e). Ki67 proliferative index was high (f).

The other 61 nodal "pure" FL (Figure 2a) stained more frequently for BCL6 (96.7%) than for CD10 (90.1%); staining for both GC-markers was detected in 53 cases (86.9%; Figure 2b, c). Cytoplasmic positivity for HGAL was found both in the nodular and in interfollicular components of FL (Figure 2d). In the remaining cases BCL6 was positive in 6 and CD10 in two. BCL2 positivity was found in 53 cases (86.9%): it ranged from faint and partial, limited to a thin perinuclear distribution and recognizable only at higher magnification (Figure 2e), to strong and diffuse, visible at glance at low power. Ki67-antigen proliferative index was highly correlated with grade (ANOVA test, p < 0.001; Figure 2f). Molecular tests were positive in 49 cases (80.3%): in 14 both *IGH@ *gene rearrangement and *MBR-BCL2/JH *rearrangement were positive, in the remaining 35 only the test of clonality was positive.

In 10 cases of nodal FL, there was a variable admixture of LBCL, displayed by the presence of a diffuse interfollicular infiltrate of cohesive large blasts. For the purposes of the present study, evaluation of immunostainings in these cases was done only in the areas considered as FL which mainly coincided with follicular areas highlighted by networks of dendritic follicular cells staining either with CD21 or CD23; only in one case there was also a diffuse areas of FL without cohesive blast clusters, not realizing the pattern of LBCL. Therefore the pattern in these cases was mainly nodular. FL cells stained positively for BCL6 and BCL2 in 9 cases and for CD10 in 8. HGAL was positive in all cases. Mean Ki67-antigen proliferative index was significantly higher than in "pure" FL (68.0 vs 31.82; ANOVA test, p < 0.0001). Molecular tests were positive in 9 cases (90%): in one case both *IGH@ *gene rearrangement and *MBR-BCL2/JH *rearrangement were positive, in the remaining 8 only test of clonality was positive.

In CFL all cases showed staining for CD10, BCL6, and HGAL, and 4 for stained for BCL2. Ki67 proliferative index was lower than that of FL combined with LBCL (ANOVA test, p < 0.001). Only monoclonal IGH@ gene rearrangement was detected in 4 cases.

The percentage of HGAL-positive cells ranged from 5 to 100% (mean, 64.89 ± 21.10). The number of neoplastic cells stained for HGAL was similar between CFL, FL and FL combined with LBCL. Applying the cut-off of 20%, 80 cases were considered positive (97.6%) and 2 (2.4%) negative. Immunostaining for HGAL was more frequently positive than that for BCL6 and CD10; the cases negative for BCL6 (5) or for CD10 (7) or for both marker (1) were all positive for HGAL, whereas the two cases negative for HGAL were positive with BCL6. Combination of positivity of the three GC-marker is reported in Table 2.

**Table 2:** Combination of GC-marker immunoreactivity in the series of FL

	Triple-positive N.cases (%)	Double-positive N.cases (%)		Single-positive N.cases (%)	
**Groups (N.cases)**	**HGAL/BCL6/CD10**	**HGAL/BCL6**	**HGAL/CD10**	**BCL6**	**HGAL**

**In situ FL (6)**	3 (50%)	-	3 (50%)	-	-

**Nodal FL (61)**	53 (87.1%)	4 (6.5%)	2 (3.2%)	2 (3.2%)	-

**FL+LBCL (10)**	8 (80%)	1 (10%)	-	-	1 (10%)

**CFL (5)**	5 (100%)	-	-	-	-

**Total (82)**	69 (84.2%)	5 (6.1%)	5 (6.1%)	2 (2.4%)	1 (1.2%)

## Discussion

Immunohistochemical analysis is a fundamental aid to morphological examination in the diagnosis of FL and relies on identification of either CD10 or BCL6 as GC-markers, and of BCL2 expression as equivalent of the typical translocation involving *BCL2 *gene. Normal polarization loss and abnormal Ki67-antigen proliferative index, particularly a low index, are both parameters useful in FL distinction from hyperplastic GCs that can be present in reactive hyperplasia and in other lymphomas arising in the mantles or in the marginal zone obliterating normal GCs. The immunostaining elicits also the recognition as "in situ" follicular neoplasm of germinal centers with atypical appearance [3]. *IGH@*-*BCL*2 gene translocation detection is not typically required for diagnosis, as the combined morpho-phenotypic analysis is sufficient in most cases. HGAL gene protein has been proposed as an adjunctive GC-marker which can be detected in benign and malignant follicles [4,15,16]. In our FL series HGAL was more frequently positive (97.6%) than that BCL6 (92.7%) and CD10 (90.2%); the cases negative for bcl6 and/or for CD10 were all positive for HGAL, whereas the two cases negative for HGAL were positive with BCL6; no difference in HGAL immunostaining was found among different malignant subtypes or grades. Previous studies have provided data also on HGAL immunostaining in lymphoma tissues utilizing a specific monoclonal antibody and the tissue microarray technology: in the study by Natkunam et al. [16] HGAL was found in a percentage in line with our results (96.2%). In the subsequent study by Weinberg et al. [21] on nodal, extranodal and primary FL, HGAL immunostaining was found in 98% of cases and in similar proportion in all subtypes; more recently, Younes et al. have reported a sensitivity of 94% in their series of FL, stressing the superiority of this marker over the others to stain higher grade and the interfollicular and diffuse areas [4]. In cutaneous primary B-cell lymphomas HGAL also proved to be very useful in the distinction between GC-derived and leg-type lymphomas [17]. Compared to HGAL, CD10 has some limitations due to frequent negativity in FL with high grade or with marginal differentiation [4,22,23]. BCL6 seems to be a more reliable GC-marker than CD10, as it is conserved in higher grade and in the interfollicular and diffuse areas [24,25]. Our results with CD10 and BCL6 are similar to those reported by other studies in which BCL6 was expressed in 94-98% of FL whereas CD10 was expressed in 78-89% [21,22]. Another study, however, reported a lower expression for BCL6 (47%) than for CD10 (77%) [4]. By using only CD10, one could run a potential risk to misdiagnosing FL as nodal marginal zone lymphoma (MZL): in their series of originally diagnosed nodal MZLs based on CD10 negativity, Salama et al. correctly reclassified 3% of them as FL by applying other GC markers, including BCL6 and HGAL [26].

By considering literature data and our present findings, we suggest a rational cost-benefit approach to the phenotyping of FL by changing the historical panel of BCL6 and CD10. By using only one GC-marker, either CD10, BCL6 or HGAL, together with BCL2, we could have the probability to miss GC-phenotype in less than 10% of FL. As the probability seems to be lower for HGAL (2.4%) than for BCL6 (7.6%) and CD10 (9.7%), all cases could be tested initially with HGAL, thus obtaining similar results as the two marker-approach with CD10 and BCL6. Only in cases negative or doubtful for HGAL, BCL6 and CD10 could be added to exclude definitively lymphoma GC. This approach would be useful in several situations, in particular when FL should be differentiated from MZLs with follicular colonization and disruption of the GC, as a commercially available marker for marginal zone differentiation is still lacking [27,28]. The interpretation of HGAL immunostaining in these circumstances seems to be easier than that of BCL6 because the latter is expressed by follicular T-helper lymphocytes. Another marker that does not stain T cells and might be useful in this setting as HGAL is the LIM-only transcription factor 2 (LMO2) [29]; according to a recent study [4], this marker, although nuclear and easily identifiable, seems to be less sensitive than HGAL (70% vs 98%), but in other studies the two markers seems comparable [21].

Nevertheless, we should always be aware of the potential pitfalls represented by the aberrant HGAL expression displayed by other non FL lymphomas. As a matter of fact, although a normal counterpart with a typical phenotype is postulated for most lymphomas, the antigenic expression may overlap rarely among different types of lymphomas. As CD10 and BCL6, also HGAL can be expressed aberrantly in MZL and in MCL. In the first description of HGAL immunostaining, Natkunam et al. [16] already observed that small subset of lymphomas not considered to be of GC derivation also showed immunostaining for HGAL, like precursor B-acute lymphoblastic lymphoma, splenic and nodal MZL, and plasma cell neoplasms. Furthermore, because nodal MZLs are thought to arise from histogenetically heterogeneous subgroups of marginal zone cells, it is not surprising that a subset of these lymphomas express GC markers such as BCL6 and HGAL. However, together with morphologic features and other differentially expressed immunohistologic markers such as CD10 and coexpression of CD43, HGAL staining is likely to be useful in separating FLs from MZLs. Difficult cases can be finally solved by molecular demonstration of the typical translocation of FL, particularly when the differential diagnosis is with MZLs. Despite any possible limitations of specimen age and fixation method, a higher success rate is achievable by FISH (56-100%) than by PCR (30-83%), because FISH is capable of detecting breakpoints that lie outside the regions covered by the PCR strategy and because absolute sequence complementarity is not required [8]. Our series well confirms the PCR limitations, as we found a PCR detection rate about 30% utilizing primers detection only MBR breakpoints; the low sensitivity would be increased by detecting other less frequent breakpoints with multiplex PCR.

## Conclusions

HGAL can be used in the immunostaining of FLs as the most sensitive germinal center (GC)-marker; when applied alone, it would reduce the immunostaining costs, reserving the use of the other two markers only to HGAL-negative cases.

## Competing interests

The authors declare that they have no competing interests.

## Authors' contributions

GG have contributed to study conception and design, to the acquisition of data, to the analysis and interpretation of data and was involved in drafting the manuscript. GL, AZ and AC have contributed to the acquisition of data and to the revision of the manuscript. FG and DS have contributed to the analysis, interpretation of data and to the revision of the manuscript. CR and PL have contributed to the study design and to the revision of the manuscript. All authors read and approved the final manuscript.
